# Multiple Myeloma With Retroperitoneal Extramedullary Plasmacytoma Causing Renal Failure and Obstructive Shock From Inferior Vena Cava Compression: A Case Report

**DOI:** 10.7759/cureus.31056

**Published:** 2022-11-03

**Authors:** Usman Ilyas, Zaryab Umar, Amee M Pansuriya, Abrahim Mahmood, Ricardo Lopez

**Affiliations:** 1 Internal Medicine, Icahn School of Medicine at Mount Sinai, Queens Hospital Center, Jamaica, USA; 2 Internal Medicine, New York Institute of Technology College of Osteopathic Medicine, Old Westbury, USA

**Keywords:** plasmacytoma, point-of-care ultrasound, obstructive renal failure, inferior vena cava compression, multiple myeloma treatment, obstructive shock, extramedullary multiple myeloma

## Abstract

Multiple myeloma (MM) is a hematologic malignancy of plasma cells. It can lead to multiorgan involvement and thus may present with various clinical manifestations, the most common being hypercalcemia, renal impairment, anemia, and bone involvement. Extramedullary multiple myeloma (EM) with soft tissue plasmacytoma (plasma cell tumor of soft tissue outside of the bone marrow) is an uncommon finding in patients with MM, which, when present, is clinically symptomatic based on the location of the tumor. An EM in the retroperitoneum is an infrequent presentation with only a few reported cases. We present a 56-year-old male with a history of retroperitoneal EM near the renal pelvis causing renal failure, presenting with obstructive shock from inferior vena cava (IVC) compression. Perirenal retroperitoneal plasmacytoma causing acute renal failure has been previously reported. A retroperitoneal EM plasmacytoma compressing the IVC precipitating obstructive shock is a unique finding that has yet to be mentioned in the literature. This report hopes to highlight the consideration of EM in patients with MM with obstructive symptoms, particularly in patients such as ours, who had a history of EM in the past. In addition, it shows the utility of bedside point-of-care ultrasound (POCUS) in the intensive care unit. After seeing persistently collapsed IVC on POCUS despite aggressive fluid management with worsening lower extremity edema and ascites, a presumptive diagnosis was made and later confirmed with magnetic resonance imaging (MRI). Although IVC stenting was planned initially, it was deferred due to fear of stent migration after chemotherapy. He was initially stabilized with vasopressors and treated with chemotherapy with cyclophosphamide and dexamethasone, which resolved hypotension. Timely intervention allowed vasopressors to be tapered, and he was subsequently discharged with outpatient chemotherapy.

## Introduction

Multiple myeloma (MM) accounts for about 10% of all hematologic malignancies [1]. While MM was previously considered to be a separate disease, it is now understood that it is a part of a spectrum of disorders of cytogenetically distinct plasma cell malignancies [1]. Extramedullary multiple myeloma (EM) is a less frequent manifestation of multiple myeloma, where myeloma cells infiltrate other organs and/or circulate freely in the blood after becoming independent of the bone marrow microenvironment, with symptoms related to the location of the mass [2,3]. Although superior vena cava (SVC) syndrome is a well-known entity associated with bronchogenic carcinoma and has been reported before with extramedullary plasmacytoma, inferior vena cava (IVC) compression is rare [4,5]. This case illustrates a rare etiology of obstructive shock resulting from retroperitoneal EM compressing the IVC.

## Case presentation

This case report is of a 56-year-old male with a past medical history of hypertension, hyperlipidemia, cardiomyopathy, benign prostatic hyperplasia, and multiple myeloma metastatic to the ribs. Multiple myeloma was diagnosed initially in late 2019 with a sternal marrow and rib biopsy at a different hospital, which showed monoclonal lambda-positive plasma cells with diffuse infiltration of the marrow. In addition, the cells exhibited aberrant expression of B-cell leukemia/lymphoma 1 (BCL-1) and were positive for IgG. He was initially treated with weekly Velcade, dexamethasone, and Zometa before receiving palliative radiation therapy to the lumbar spine.

**Figure 1:**
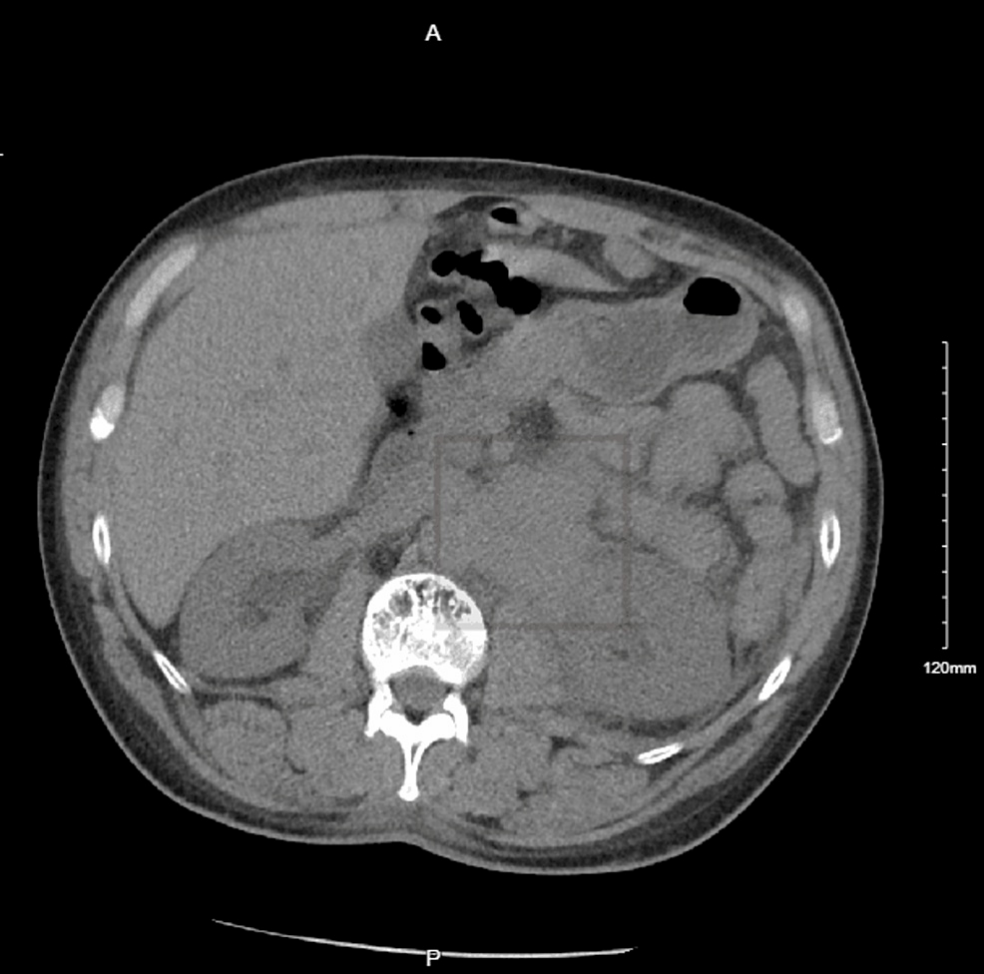
CT of the abdomen and pelvis without contrast showing confluent mass adjacent to the left side of the aorta at the level of the left kidney, poorly evaluated without intravenous contrast. CT: computed tomography

The patient was referred to our hospital for a stem cell transplant. However, he could not initially keep the appointment and was lost to follow-up for six months. On return, his initial computed tomography (CT) of the abdomen demonstrated extramedullary retroperitoneal plasmacytoma (Figure 1).

The patient was restarted on dexamethasone 20 mg orally and Velcade 1.3 mg/m^2^ before he developed coughing with streaks of blood. Over the next few days, he developed shortness of breath, progressively worsening. Hence, he presented to the emergency department and was intubated for respiratory failure. After the bronchoscopy was negative for infection, his respiratory failure was attributed to heart failure. Since his ejection fraction (EF) was preserved at 50% with normal right ventricular systolic function, diastolic dysfunction was suspected; however, the technetium nuclear medicine scan was negative for transthyretin amyloidosis, and therefore, the etiology of cardiomyopathy was attributed to either bortezomib chemotherapy or amyloid light-chain (AL) amyloidosis.

**Figure 2:**
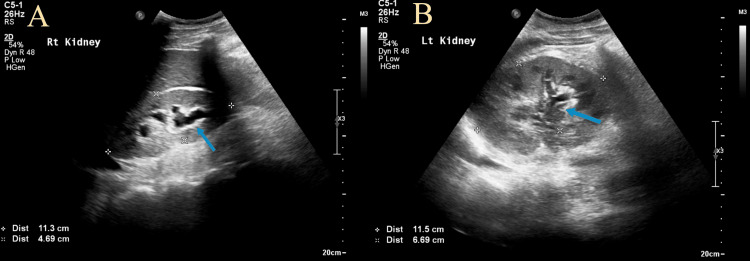
Ultrasound of the kidneys demonstrating bilateral hydronephrosis (blue arrows) with (A) the right kidney measuring 11.3 cm by 4.7 cm and (B) the left kidney measuring 11.5 cm by 6.7 cm.

Two weeks later, before his next follow-up appointment, he received a call back from the laboratory for potassium of 7 mmol/L (normal range: 3.5-5.1 mmol/L) with an increase in creatinine to 8 mg/dL (normal range: 0.50-1.20 mg/dL) from a baseline of 1.5 mg/dL. Ultrasound of the kidneys demonstrated bilateral hydronephrosis (Figure 2). Renal pelvis compression from the prior known mass was suspected.

**Figure 3:**
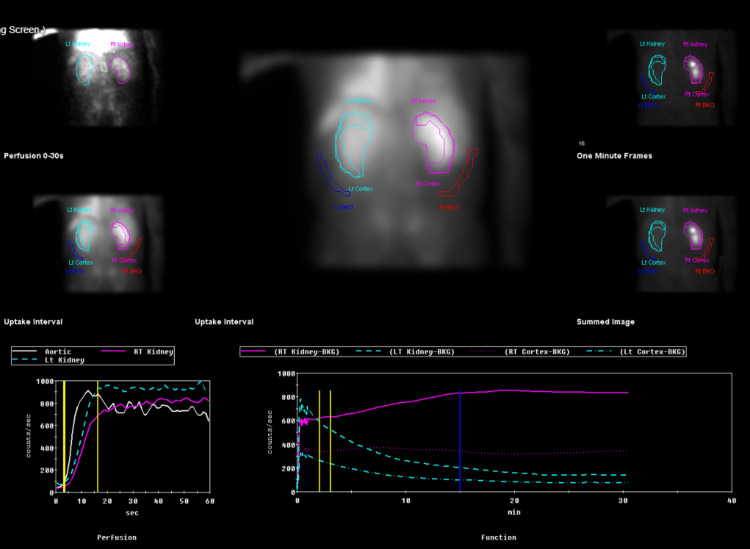
Nuclear medicine renal morphology scan showing decreased uptake within the enlarged left kidney and minimal function noted visually. Split function values are within normal limits, indicating good flow to both kidneys. The right kidney demonstrates a continuous uprising renogram curve, and findings are concerning for obstruction, with no significant response to Lasix noted.

Interventional radiology-guided bilateral nephrostomy tubes were placed with an improvement in renal function and electrolyte abnormalities. However, a nuclear medicine renal morphology scan showed minimal left renal function (Figure 3), so the left-sided nephrostomy tube was removed.

**Figure 4:**
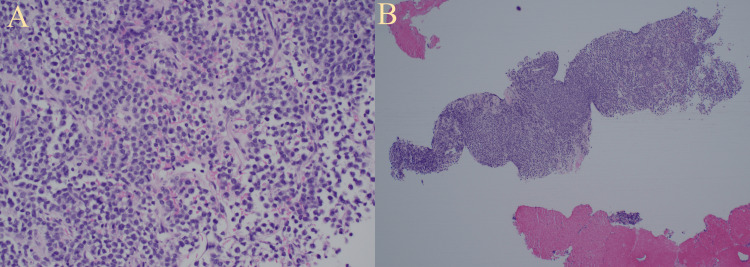
Biopsy of the retroperitoneal mass. (A) Hematoxylin and eosin staining of the retroperitoneal mass at high power. An atypical proliferation of lymphocytoid cells with variable cytoplasm and occasional nucleoli associated with necrosis, along with focal areas with eosinophilic materials, are noted. (B) Sheets of abnormal myeloma tumor cells are visible at low power.

**Figure 5:**
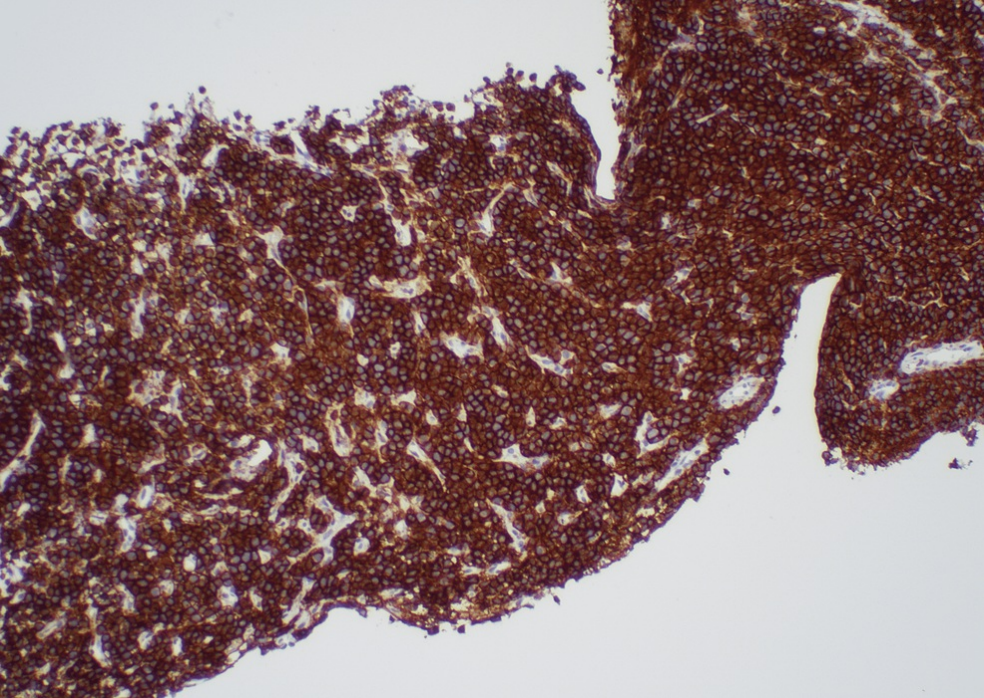
Immunostaining of the retroperitoneal mass showing neoplastic cell positivity for CD138. CD138: cluster of differentiation 138

**Figure 6:**
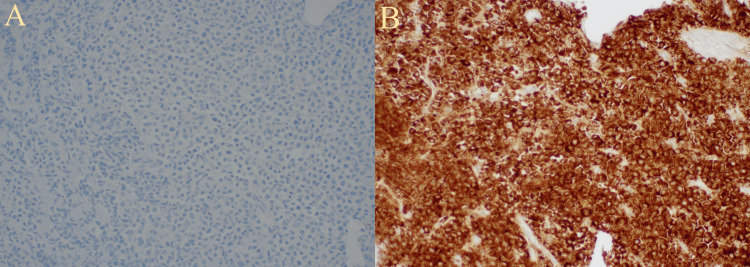
Stains for kappa and lambda of the mass showing (A) minimal staining pattern for kappa and (B) excess lambda staining.

A biopsy of the retroperitoneal mass showed neoplastic plasma cells with necrosis (Figure 4). Immunostaining demonstrated neoplastic cell positivity for cluster of differentiation 138 (CD138) and negativity for cytokeratin and synaptophysin (Figure 5). Stains for kappa and lambda showed excess staining for lambda (Figure 6).

Although he was subsequently discharged for planned outpatient chemotherapy, possibly daratumumab with either immunomodulatory drugs or cyclophosphamide, in his revisit to the clinic, he was found to be hypotensive at 80/50 and tachycardic with a heart rate (HR) of 127 beats/minute. He was referred to the emergency department (ED), complaining of loss of appetite, nausea, vomiting with each feed, and constipation with one bowel movement every week. The dysphagia was worse with a solid diet as he was vomiting shortly afterward and had early satiety with a small quantity of feed. He also had skipped his oral medications for the last three days, including furosemide, amlodipine, and metoprolol, as he was feeling weak and dizzy and also partially in the setting of inability to tolerate oral medication. His physical examination showed no jugular venous distention, protuberant belly, tenderness to deep palpation at the epigastrium, and 3+ bilateral lower extremity edema. Laboratory results on admission are shown in Table 1. Urinalysis and urine culture reports are shown in Table 2.

**Table 1:** Initial laboratory results of the patient on admission. ALT: alanine transaminase, AST: aspartate transaminase, ALP: alkaline phosphatase, TB: tuberculosis

Laboratory parameter	Patient’s value	Reference range
Hemoglobin level	6.9 g/dL	12-16 g/dL
White blood cell count	4.29 × 10^3^/mcL	4.80-10.80 × 10^3^/mcL
Platelets	540 × 10^3^/mcL	150-450 × 10^3^/mcL
Sodium	127 mmol	136-145 mmol
Potassium	5.5 mmol/L	3.5-5.1 mmol/L
Bicarbonate	17 mmol/L	22-29 mmol/L
Blood urea nitrogen	46 mg/dL	6-23 mg/dL
Creatinine	4.72 mg/dL	0.50-1.20 mg/dL
ALT	17 U/L	0-33 U/L
AST	55 U/L	5-32 U/L
ALP	63 U/L	35-104 U/L
Prothrombin time	13.2 seconds	10-13 seconds
QuantiFERON-TB Gold Plus	Negative	Negative
Blood cultures	Negative	Negative
Serum lactate	2.2 mmol/L	0.6-1.4 mmol/L
Immunoglobulin free light chains (blood)		
Immunoglobulin kappa	6.1 mg/dL	0.33-1.94 mg/dL
Immunoglobulin lambda	113.39 mg/dL	0.57-2.63 mg/dL
Kappa/lambda ratio	0.05	0.26-1.65

**Table 2:** Urinalysis and urine culture reports of the patient on admission.

Laboratory parameter	Patient’s value	Reference range
pH	6.0	5.0-7.5
Appearance	Turbid	Clear
Blood	Large	Negative
Protein	300 mg/dL	Negative
Nitrite	Negative	Negative
Leukocyte esterase	Moderate	Negative
White blood cells	>50	0-4 high power field
Red blood cells	>50	0-3 high power field
Bacteria	Many	Negative
Squamous epithelial cells	0-4	0-4 high power field
Urine culture	No growth	No growth

**Figure 7:**
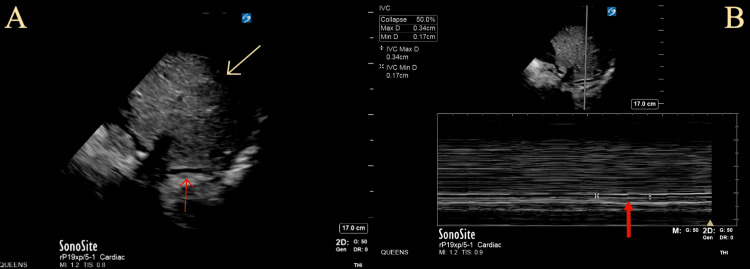
Point-of-care ultrasound with (A) subxiphoid view demonstrating the inferior vena cava (red arrow) and liver (yellow arrow), and (B) collapsed inferior vena cava (red arrow) with a maximum and minimum diameter of 0.34 cm and 0.17 cm, respectively.

Initial assessment included septic shock due to urinary tract infection from a right-sided nephrostomy tube and failure to thrive in the setting of decreased oral intake and multiple myeloma. He was started on vancomycin and meropenem along with stress doses of steroids, and fluid boluses were given. However, he remained persistently hypotensive with mean arterial pressure below 65 mmHg. Therefore, he was started on a norepinephrine drip and transferred from the emergency department to the intensive care unit for further care. He failed to respond to fluid boluses, antibiotics, and stress steroids. Midodrine, given to taper Levophed, also failed. Over the next few days, his feeding and vomiting worsened, and he developed worsening bilateral lower limb pitting edema. The bedside point-of-care ultrasound (POCUS) showed a persistent collapsed inferior vena cava despite 2 L of Ringer’s lactate administration (Figure 7).

**Figure 8:**
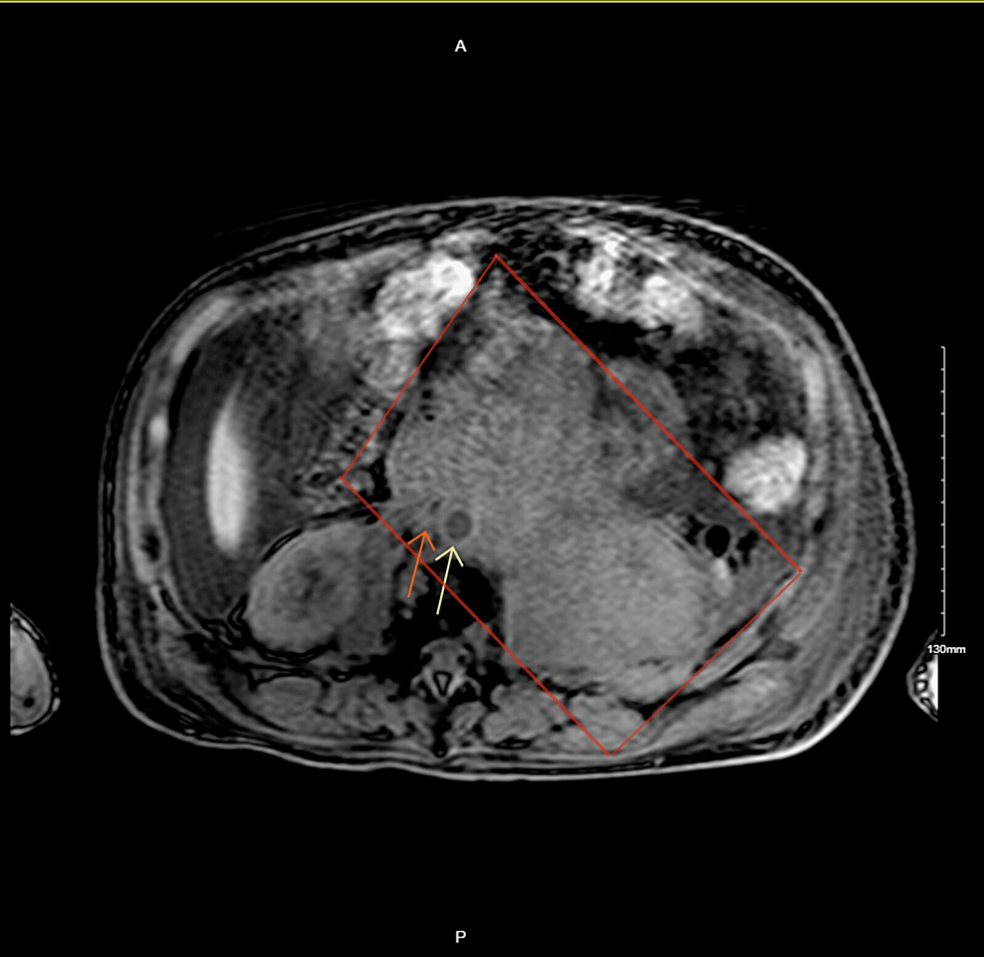
MRI of the abdomen without contrast showing a large infiltrative left retroperitoneal mass. It surrounds the aorta (yellow arrow), and there is a narrowing of the lumen of the inferior vena cava (orange arrow), indicating a mass effect. MRI: magnetic resonance imaging

Given his prior history of retroperitoneal plasmacytoma, inferior vena cava obstruction by the mass was suspected. Thus, an abdominal magnetic resonance imaging (MRI) was ordered that confirmed the suspicion of obstructive shock secondary to inferior vena cava compression by the retroperitoneal plasmacytoma (Figure 8).

Stress-dose steroids were discontinued, and high-dose dexamethasone 40 mg daily for four days and one dose of cyclophosphamide 550 mg were given as per oncology. Although the plan was to discontinue Levophed with the initiation of chemotherapy once the patient was hemodynamically stable, persistent attempts to taper off norepinephrine failed. Vascular surgery was consulted and advised placement of an inferior vena cava stent. The patient was transferred to another facility for inferior vena cava stenting. However, ultimately, stent placement was not done as the risk for stent migration was deemed high after the response of the retroperitoneal plasmacytoma to chemotherapy and subsequent decrease in size. Since his renal function improved, a trial of cyclophosphamide, bortezomib, and dexamethasone (CyBorD) was given, which was successful. He was discharged with continued outpatient chemotherapy.

Over the next year, his clinical course briefly included initial therapy with daratumumab, carfilzomib, lenalidomide, and dexamethasone. However, he had progression of the disease with four cycles of treatment and was admitted with gastric/duodenal obstruction due to worsening retroperitoneal mass. Dexamethasone, cyclophosphamide, etoposide, and cisplatin were initiated, but secondary to delayed chemotherapy in the setting of COVID-19, his disease progressed further with hospitalization for bowel obstruction. He received treatment with dexamethasone, cyclophosphamide, etoposide, and cisplatin (VD-PACE) with relief of symptoms. He was not deemed a good candidate for a chimeric antigen receptor T-cell (CAR-T) therapy trial. As a last resort, he is on belantamab mafodotin, having received one cycle of therapy.

## Discussion

Obstructive shock is rare, with a relative incidence of 1%-2% compared to other shock causes [6]. The well-known causes of obstructive shock include pulmonary embolism, tension pneumothorax, and cardiac tamponade [7]. Abdominal compartment syndrome, cardiovascular compression from space-occupying lesions, critical aortic stenosis or coarctation, and high positive pressure ventilation states are lesser-known etiologies [8]. Although presenting signs and symptoms can vary, a common feature for most etiologies is rapid hemodynamic deterioration [8].

Extramedullary plasmacytomas are not uncommon in patients with multiple myeloma, with an incidence of 7%-18% at the time of diagnosis and up to 20% at relapse [9]. Myeloma patients with extramedullary disease usually present with symptoms related to the mass location [3]. Vanquaethem et al. reported a 79-year-old patient with SVC syndrome from extramedullary plasmacytoma compression [5]. In our case, the patient had a unique presentation of retroperitoneal plasmacytoma that not only produced obstructive symptoms leading to kidney failure requiring bilateral nephrostomy tube placement but also led to IVC obstruction causing obstructive shock. To the best of our knowledge, EM with IVC compression has not been reported in the literature. Our case highlights the challenges associated with differentiating between different forms of shock. Initially, distributive shock from urosepsis was considered, given bilateral nephrostomy tubes with a history of urinary tract infection. Only after multiple fluid boluses showing the persistent collapse of the inferior vena cava on the point-of-care ultrasound (POCUS) combined with lower extremity edema and prior history of known retroperitoneal mass, IVC syndrome was suspected and later confirmed with MRI, as the IVC was visible as a compressed slit-like structure.

The diagnosis of IVC syndrome is based mainly on clinical features, with signs of shock accompanied by lower extremity edema and radiological confirmation [4]. Imaging modalities include CT of the abdomen, which can reveal intracaval thrombi, and MRI with angiography [4]. Definitive management for obstructive shock is directed toward treating the underlying physical obstruction to blood flow [6]. In IVC syndrome, treatment commonly includes stenting or chemoradiotherapy [4]. Although IVC stent placement was initially considered, it was deferred due to fear of stent migration after the initiation of chemotherapy. The patient was able to wean off vasopressors after a high dose of dexamethasone and cyclophosphamide.

## Conclusions

IVC syndrome is a medical emergency that requires immediate recognition and urgent treatment, with therapeutic strategies specifically tailored for each patient, depending on the etiology of the compression. Obstructive shock should be kept on the differential in patients with known retroperitoneal mass. Point-of-care ultrasound is a valuable tool in the initial assessment of critically ill patients in the intensive care unit.
